# Clinical study of systemic chemotherapy combined with bronchoscopic interventional cryotherapy in the treatment of lung cancer

**DOI:** 10.1186/s12885-020-07444-6

**Published:** 2020-11-11

**Authors:** Feng Xu, Jian Song, Beizheng Xu, Jiang Wang, Jianjun Mao, Haiyan Liu, Xuanmei Li, Aibing Deng

**Affiliations:** 1 Department of Pulmonary and Critical Care Medicine, Cangzhou People’s Hospital, Cangzhou, 061000 China; 2 Tianjin Medical Uniersity, Tianjin, 300070 China

**Keywords:** Systemic chemotherapy, Bronchoscope, Interventional cryotherapy, Lung cancer

## Abstract

**Background:**

This study is designed to investigate the clinical value of systemic chemotherapy combined with bronchoscopic interventional cryotherapy in the treatment of lung cancer.

**Methods:**

A total of 412 lung cancer patients admitted to Cangzhou People’s Hospital from March 2018 to March 2020 were collected and divided into test group and control group based on their treatment schedules. The test group received systemic chemotherapy combined with bronchoscopic interventional cryotherapy, while the control group received systemic chemotherapy alone. Tumor objective response rate (ORR), disease control rate (DCR), serum tumor marker levels, serum matrix metalloproteinase (MMP) content, T cell subset level, survival time and adverse reactions of the two groups were observed.

**Results:**

The ORR and DCR of the test group were better than those of the control group, while those of the non-small cell lung cancer (NSCLC) patients in the test group were better than patients with small-cell lung cancer (SCLC) (*P* <  0.05). There was no significant difference in serum tumor marker levels, MMP content and T cell subset level between the two groups before treatment. After treatment, the serum tumor marker levels along with serum MMP-2, MMP-9 and CD8^+^ levels in the test group decreased more remarkably, while CD4^+^ and CD4^+^/CD8^+^ levels increased more significantly than those in the control group (*P* <  0.05). The serum MMP-2 and MMP-9 of NSCLC patients in the test group decreased more remarkably than those of SCLC patients, while there was no significant difference in CD8^+^, CD4^+^ and CD4^+^/CD8^+^. The progression-free survival and overall survival of the test group were obviously longer than those of the control group. The same trend was observed in NSCLC patients compared with SCLC patients in the test group (*P* <  0.05).

**Conclusions:**

Systemic chemotherapy combined with bronchoscopic interventional cryotherapy for lung cancer has good clinical efficacy and safety, and can be widely used in clinical practice.

## Highlights

1. The curative effect of systemic chemotherapy combined with bronchoscopic interventional cryotherapy is superior than systemic chemotherapy alone;
2. Systemic chemotherapy combined with bronchoscopic interventional cryotherapy can significantly prolong the survival time of patients;
3. Systemic chemotherapy combined with bronchoscopic interventional cryotherapy is more effective in patients with non-small cell lung cancer.

## Background

Lung cancer is a prevalent malignant tumor in clinical practice with high and annually increasing morbidity and mortality worldwide [1, 2]. Lung cancer is easy to be neglected due to a lack of obvious symptoms in the early stage. The disease has developed to the middle and advanced stage when patients appear obvious discomfort, which makes patients miss the opportunity of surgery. Systemic chemotherapy is the main treatment for patients with advanced lung cancer, which can effectively kill tumor cells to prolong the survival time of patients, but there are many side effects and patients will suffer greatly. Compared with other cancer patients, lung cancer patients tend to bear more severe symptom burdens related to the disease itself and treatment in the process of disease development and treatment [3], and these symptoms seriously affect life quality and functional status of patients. Therefore, it is crucial to optimize the treatment for lung cancer patients.

Interventional cryotherapy is a non-surgical treatment for tumor patients in recent years, therapeutic mechanism of which includes the following aspects: (1) In terms of physical changes, ice crystals are produced in tissues by cryogenic freezing. Intracellular ice crystals lead to intracellular dysfunction, and extracellular ice crystals cause intracellular dehydration, thereby leading to cell disintegration and death, which is the main cause of cell death. (2) In terms of chemical changes, freezing can change pH value, destroy cell protein, enzyme system, cell metabolism and cause cell death. (3) Vascular effects include slow stasis of blood flow, red cell agglutination, vascular wall destruction, capillary embolism, and local tissue necrosis due to ice crystals blocked in micro vessels. Highly hydrated tissues are sensitive to cryotherapy. (4) In terms of immune functions, basic and clinical studies have confirmed that cryotherapy can significantly improve the contents of OKT3+ and OKT4+ and the ratio of OKT3+/OKT4+ in immune cells, significantly increase the expression of IL-2R, and improve the immune function of patients [4, 5]. Cryotherapy has been widely used in various diseases in clinic with good outcomes, including prostate cancer [6], irreversible pulpitis [7], esophageal cancer [8], cervical cancer [9]. But the efficacy of cryotherapy in combination with systemic chemotherapy in lung cancer patients is unclear.

Herein, 412 patients diagnosed with lung cancer who were admitted to Cangzhou People’s Hospital from March 2018 to March 2020 were randomly divided into two groups according to their main treatment schedules during hospitalization. The clinical efficacy and survival time of patients after treatment with cryotherapy combined with traditional systemic chemotherapy were investigated. In addition, we also studied the effect of cryotherapy combined with systemic chemotherapy on different types of lung cancer including non-small cell lung cancer (NSCLC), small cell lung cancer (SCLC), lung adenocarcinoma (LADC) and lung squamous cell carcinoma (LSCC).

## Methods

### Data collection

Inclusion criteria were as follows: (1) The Karnofsky performance status (KPS) score of patients was above 60. (2) Life expectancy was greater than 6 months. (3) Patients were older than 18. (4) There was no significant trachea function damage in heart, kidney, liver, spleen and stomach. (5) The patients had not received any chemoradiotherapy or surgical treatment before diagnosis. (6) All patients signed written informed consent.

According to the inclusion criteria, a total of 412 lung cancer patients (including 259 males and 153 females, aged 39–82) admitted to Cangzhou People’s Hospital from March 2018 to March 2020 were involved in this study. All the patients were diagnosed with lung cancer by imaging and pathological diagnosis after admission. They were divided into test group (*n* = 208) and control group (*n* = 204) according to their treatment schedules during hospitalization. The test group received systemic chemotherapy combined with bronchoscopic interventional cryotherapy, while the control group received systemic chemotherapy alone. General clinical data of all the patients consisting of age, gender, smoking history, KPS score, disease staging, classification, and metastasis were listed in Table 1.

**Table 1:** General clinical data of all the patients

Characters	Test group (***n*** = 208)	Control group (***n*** = 204)
Age group, years		
< 60	96 (46.2%)	95 (46.6%)
≥ 60	112 (53.8%)	109 (53.4%)
Gender		
Male	135 (64.9%)	124 (60.8%)
Female	73 (35.1%)	80 (39.2%)
Smoking history		
Never smoker	29 (13.9%)	23 (11.3%)
Former smoker	81 (39.0%)	88 (43.1%)
Current smoker	98 (47.1%)	93 (45.6%)
KPS score		
60–75	80 (38.5%)	84 (41.2%)
> 75	128 (61.5%)	120 (58.8%)
Metastasis		
Yes	99 (47.6%)	96 (47.1%)
No	109 (52.4%)	108 (52.9%)
Disease staging		
I-II	113 (54.3%)	106 (52.0%)
III-IV	95 (45.7%)	98 (48.0%)
Classification		
SCLC	44 (21.2%)	40 (19.6%)
NSCLC	164 (78.8%)	164 (80.4%)
NSCLC type		
LADC	109 (66.5%)	108 (65.9%)
LSCC	55 (33.5%)	56 (34.1%)

### Therapeutic schedules

#### Systemic chemotherapy

All the patients were given 1000–1250 mg/m^2^ gemcitabine (Jiangsu Aosaikang Pharmaceutical Co., LTD., State Food and Drug Administration (SFDA) No. HB20093698) by intravenous infusion for 30 min on day 1 and day 8. Then, 25 mg/m^2^ cisplatin (Yunnan BIOVALLEY Pharmaceutical Co., LTD., SFDA No. H20043888) was given by intravenous infusion on the first 1–3 days. Next, 250 mg gifitinib (AstraZeneca Pharmaceutical Co., LTD.; SFDA No. J20140471) was given by oral. Patients had to receive at least 2 courses of treatment with 21 days for one course.

#### Bronchoscopic interventional cryotherapy

On the basis of systemic chemotherapy, patients in the test group received bronchoscopic interventional cryotherapy. The specific plan was as below: Preoperative fasting for 4 h. Electronic bronchoscopy was firstly performed to identify tumor location, size, and degree of bronchial obstruction. Aerosol inhalation of 2% lidocaine was used for anesthetization. After entering the operating room, patients went through electrocardiogram, oxygen saturation and non-invasive blood pressure. K300 cryotherapy apparatus was used to freeze tumor tissues, and a metal probe sterilized with alcohol was inserted to tumor lesions along the bronchoscope. The tumor had whitish dehydration after 2 min, then freezing was stopped for naturally melting, with one freezing and thawing cycle for 3 ~ 4 min. For larger tumors, multidirectional cryopreserved options were available.

### Evaluation indexes

1. Serum tumor marker levels: peripheral venous blood was collected from the two groups before and after different treatments. The levels of carcino-embryonic antigen (CEA), neuron-specific enolase (NSE), cytokeratin fragment antigen (CYFRA21-1) and carbohydrate antigen 199 (CA199) were measured using the radioimmunoassay kit (Cis, France).
2. Serum MMP content and T cell subset level: peripheral venous blood was extracted from the patients after different treatments. MMP-2 and MMP-9 levels were determined by enzyme-linked immunosorbent assay (ELISA) and real-time quantitative PCR (qRT-PCR). The levels of T cell subsets, including CD4^+^, CD8^+^ and CD4^+^/CD8^+^, were determined by flow cytometry (FCM).
3. Short-term effects: the primary indexes include tumor objective response rate (ORR) and disease control rate (DCR). Short-term effects were assessed in accordance with RECIST 1.1, revised in 2009, including: Complete Remission (CR): all target lesions disappear, and short diameter of all pathological lymph nodes (both target and non-target) must be reduced to < 10 mm; Partial Remission (PR): the sum of target lesion diameters is at least 30% less than the baseline level; Stable Disease (SD): the degree of target lesion is between PR and PD; Progressive Disease (PD): the sum of target lesion diameters should be increased by at least 20% based on baseline value (if the baseline value was minimal). In addition, the absolute value of the sum of diameters must be increased by at least 5 mm (the presence of one or more new lesions is also considered as PD).

### Follow-up visit

All patients were followed up, including hospitalization and telephone follow-up. The primary assessment indexes were progression-free survival (PFS) and overall survival (OS). By March 31, 2020, all patients were followed up for 3–24 months, of which 12 patients were lost to follow-up, with a total follow-up rate of 97.1%. PFS is the time from the beginning of treatment to the presence of recurrence or PD. OS is the time from the beginning of treatment to death or loss of follow-up.

### Statistics analysis

All data were analyzed using SPSS 18.0 software, and survival curves were plotted using GraphPad Prism 7.0. The measurement data were expressed as mean ± standard deviation ($\overline{\mathrm{X}}$ ±s), and *t*-test was used for analyzing difference between the two groups. The counting data were presented using percentage (%), and Chi-square test was used to verify the data. *P* <  0.05 meant that the difference was statistically significant.

## Results

### Short-term effects

The short-term effects were evaluated after two courses of systemic treatment for patients in the control group and for patients in the test group with another two-week interventional cryotherapy. The results showed that there were significant differences in ORR and DCR between the two groups (*P* <  0.05), indicating that the treatment effect of the test group was better than that of the control group (Table 2). In order to explore whether the treatment of test group had better efficacy in all types of lung cancer, we subdivided the patients and discovered that the ORR and DCR of NSCLC patients were higher than those of SCLC patients. While there was no significant difference in ORR and DCR between patients with LADC and LSCC (Table 3). From the above results, it could be concluded that systemic chemotherapy combined with bronchoscopic interventional cryotherapy was superior to chemotherapy alone, with better clinical efficacy on NSCLC patients.

**Table 2:** The short-term effects of the two groups (n, %)

Project	CR	PR	SD	PD	ORR	DCR
**Test group (*n*** **= 208**)	108 (51.9)	42 (20.2)	40 (19.2)	18 (8.7)	72.1	91.3
**Control group (*n*** **= 204**)	56 (27.5)	60 (29.4)	40 (19.6)	48 (23.5)	56.9	76.5
***P*** **Value**					0.027	0.004

Note: ORR = CR + PR, DCR = CR + PR + SD

**Table 3:** Comparison of short-term effects in patients with different subtypes of lung cancer in the test group

Project	CR	PR	SD	PD	ORR	DCR
**SCLC (*n*** **= 44**)	9 (20.5)	12 (27.3)	14 (31.8)	9 (20.4)	47.8	78.9
**NSCLC (*n*** **= 164**)	99 (60.4)	30 (18.3)	26 (15.8)	9 (5.5)	78.7	94.5
***P*** **Value**					< 0.0001	0.002
**LADC (*n*** **= 109**)	70 (64.2)	19 (17.4)	15 (13.8)	5 (4.6)	81.6	95.4
**LSCC (*n*** **= 55**)	29 (52.7)	11 (20)	11 (20)	4 (7.3)	72.7	92.7
***P*** **Value**					0.13	0.55

Note: ORR = CR + PR, DCR = CR + PR + SD

### Serum tumor marker levels between the two groups

The serum tumor markers of all the patients had no significant difference before treatment, which were decreased after corresponding treatments in two groups. The levels of serum tumor markers including CEA, NSE, CYFRA21-1 and CA199 in the test group were significantly lower than those in the control group (*P* <  0.05), as shown in Table 4. Next, we further subdivided patients in the test group and observed that the serum tumor marker levels of NSCLC patients were decreased more remarkably than those of SCLC patients (*P* <  0.05). But there was no significant difference in serum tumor markers between patients with LADC and LSCC (*P* > 0.05), as exhibited in Table 5.

**Table 4:** Serum tumor marker levels of the two groups ($\overline{\mathrm{X}}$ ±s)

Project	Test group (***n*** = 208)	Control group (***n*** = 204)	***P*** Value
**Before treatment**			
CEA (ng/ml)	96.27 ± 9.57	95.38 ± 9.21	0.34
NSE (ng/ml)	88.62 ± 7.52	88.01 ± 7.44	0.41
CYFRA21-1 (ng/ml)	37.84 ± 5.90	37.58 ± 6.00	0.66
CA199 U/ml	51.83 ± 7.53	50.69 ± 7.42	0.12
**After treatment**			
CEA (ng/ml)	22.68 ± 3.53	56.53 ± 7.51	< 0.0001
NSE (ng/ml)	25.59 ± 4.53	58.19 ± 8.63	< 0.0001
CYFRA21-1 (ng/ml)	9.88 ± 0.55	22.08 ± 2.37	< 0.0001
CA199 U/ml	27.15 ± 4.69	47.95 ± 4.56	< 0.0001

**Table 5:** Serum tumor marker levels of patients with different subtypes of lung cancer in the test group ($\overline{\mathrm{X}}$ ±s)

Project	SCLC	NSCLC	***P*** Value	LADC	LSCC	***P*** Value
**Before treatment**						
CEA (ng/ml)	96.73 ± 9.11	96.01 ± 9.31	0.65	95.54 ± 9.78	95.82 ± 9.12	0.86
NSE (ng/ml)	88.71 ± 7.43	88.61 ± 7.51	0.94	88.63 ± 7.49	88.59 ± 7.49	0.97
CYFRA21-1 (ng/ml)	37.87 ± 5.87	37.84 ± 5.90	0.98	37.82 ± 5.88	37.85 ± 5.89	0.96
CA199 U/ml	51.78 ± 7.48	51.84 ± 7.52	0.96	51.82 ± 7.52	51.85 ± 7.51	0.98
**After treatment**						
CEA (ng/ml)	24.66 ± 1.55	20.25 ± 1.1	< 0.0001	20.27 ± 1.08	20.24 ± 1.09	0.87
NSE (ng/ml)	28.15 ± 1.97	22.81 ± 1.75	< 0.0001	22.83 ± 1.73	22.79 ± 1.73	0.89
CYFRA21-1 (ng/ml)	10.13 ± 0.13	9.55 ± 0.22	< 0.0001	9.54 ± 0.21	9.57 ± 0.2	0.38
CA199 U/ml	29.22 ± 2.62	24.16 ± 1.7	< 0.0001	24.14 ± 1.72	24.17 ± 1.71	0.92

### The levels of serum MMP and T cell subsets between the two groups

No significant difference was observed in the levels of MMP (including serum MMP-2 and MMP-9) and T-cell subsets between the two groups before treatment. After treatment, the levels of MMP-2 and MMP-9 in the two groups were significantly reduced, and the decrease was more obvious in the test group (*P* <  0.05). For MMP2 and MMP9, we also detected the expression levels between the two groups before and after treatment, and the results also exhibited that the expression levels of serum MMP2 and MMP9 in the two groups were prominently decreased after treatment (*P* <  0.05) (Figure S1A-B). The CD4^+^ level in both groups was increased after treatment, with remarkable increase in the test group (Figure S2A). The CD8^+^ level in the two groups, especially in the test group, was decreased to a certain extent after treatment (Figure S2B). In addition, the CD4^+^/CD8^+^ level in the test group was increased, while that in the control group was decreased to a certain extent after treatment, indicating a significant difference between the two groups (*P* <  0.05) (Figure S2C) (Table 6). Next, we further analyzed the patients in the test group and found that the levels of MMP-2 and MMP-9 in NSCLC patients were decreased more dramatically than those in SCLC patients (*P* <  0.05), while there was no significant difference in the levels of CD8^+^, CD4^+^ and CD4^+^/CD8^+^ (Figure S2 D-F). We also detected the expression levels of MMP2 and MMP9 in NSCLC and SCLC patients, and it was showed that MMP2 and MMP9 expression levels in patients after treatment were significantly decreased (*P* <  0.05) (Figure S1C-D). Moreover, there was no significant difference in levels of MMP-2, MMP-9 and T cell subsets between patients with LADC and LSCC (*P* > 0.05) (Figures S1E-F and S2G-I) (Table 7). The results suggested that the treatment schedules in the test group was better for NSCLC patients and had similar efficacy on patients with LADC and LSCC.

**Table 6:** The levels of MMP and T cell subsets of the two groups before and after treatment ($\overline{\mathrm{X}}$ ±s)

Project	Test group (***n*** = 208)	Control group (***n*** = 204)	***P*** Value
**Before treatment**			
MMP-2 (ng/L)	182.51 ± 12.66	180.36 ± 12.92	0.09
MMP-9 (ng/L)	207.24 ± 22.50	203.31 ± 23.11	0.08
CD4^+^ %	33.15 ± 4.24	33.12 ± 4.13	0.94
CD8^+^ %	30.51 ± 3. 39	30.76 ± 3.54	0.46
CD4^+^/CD8^+^	1.17 ± 0. 25	1.18 ± 0.21	0.66
**After treatment**			
MMP-2 (ng/L)	32.51 ± 4.11	76.37 ± 6.39	< 0.0001
MMP-9 (ng/L)	34.38 ± 4.82	77.13 ± 7.09	< 0.0001
CD4^+^ %	37.82 ± 5.12	33.73 ± 4.73	< 0.0001
CD8^+^ %	27.05 ± 3.83	29.18 ± 3.10	< 0.0001
CD4^+^/CD8^+^	1.33 ± 0.21	1. 16 ± 0. 20	< 0.0001

**Table 7:** MMP content and levels of T cell subsets before and after treatment in patients with different subtypes of lung cancer in the test group

Project	SCLC	NSCLC	***P*** Value	LADC	LSCC	***P*** Value
**Before treatment**						
MMP-2 (ng/L)	182.93 ± 12.24	181.52 ± 11.67	0.48	180.22 ± 10.37	181.05 ± 12.14	0.65
MMP-9 (ng/L)	206.17 ± 21.43	207.88 ± 21.86	0.64	207.61 ± 21.59	208.20 ± 21.54	0.87
CD4^+^%	33.63 ± 3.76	33.00 ± 4.09	0.36	32.55 ± 3.64	33.17 ± 3.93	0.32
CD8^+^%	30.87 ± 3.03	30.46 ± 3.34	0.46	30.25 ± 3.13	30.77 ± 3.03	0.31
CD4^+^/CD8^+^	1.20 ± 0.22	1.15 ± 0.23	0.20	1.17 ± 0.21	1.13 ± 0.21	0.25
**After treatment**						
MMP-2 (ng/L)	35.6 ± 1.02	30.52 ± 2.12	< 0.0001	30.71 ± 2.31	30.78 ± 1.86	0.85
MMP-9 (ng/L)	35.37 ± 1.25	31.08 ± 1.52	< 0.0001	31.13 ± 1.47	30.86 ± 1.3	0.25
CD4^+^%	38.15 ± 4.97	37.78 ± 5.08	0.66	37.33 ± 4.63	37.81 ± 5.05	0.54
CD8^+^%	27.22 ± 3.66	26.93 ± 3.71	0.64	26.51 ± 3.29	27.13 ± 3.51	0.27
CD4^+^/CD8^+^	1.36 ± 0.18	1.33 ± 0.21	0.39	1.30 ± 0.18	1.34 ± 0.20	0.20

### Survival time

**Fig. 1:**
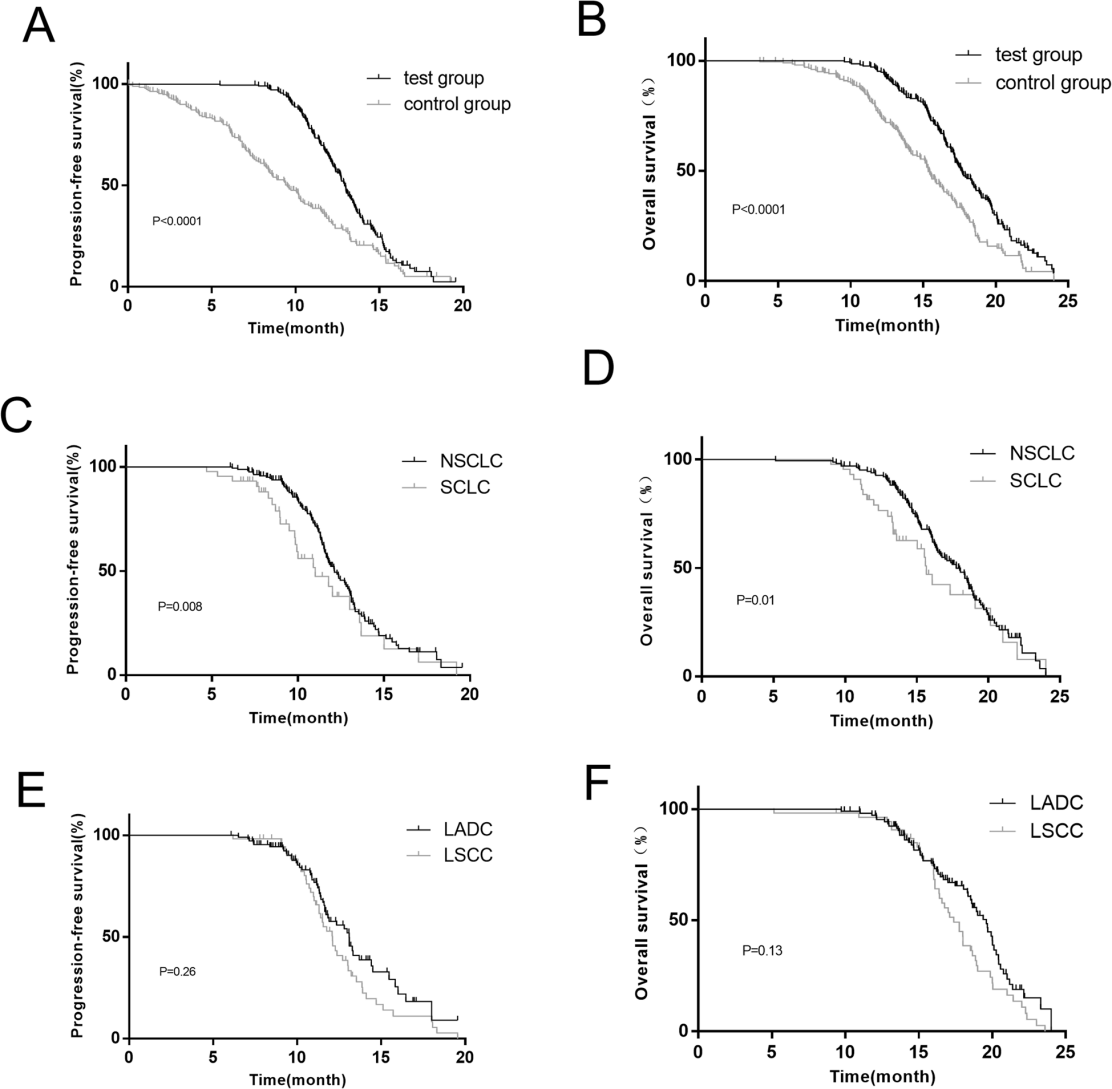
PFS and OS of patients in different groups. The PFS and OS between the test and control groups (**a**-**b**), NSCLC patients and SCLC patients in the test group (**c**-**d**), and patients with LADC and LSCC in the test group (**e**-**f**) were compared

The median follow-up time was 17.2 months for all patients. The median PFS was 13.9 months and the median OS was 17.5 months of the test group. While the median PFS was 8.3 months and the median OS was 14 months of the control group. There were significant differences in PFS and OS between the two groups (*P* <  0.05) (Fig. 1a-b). Furthermore, the PFS and OS of NSCLC patients in the test group were longer than those of SCLC patients, and the PFS and OS of LADC patients were similar to those of LSCC patients, with no significant difference (*P* > 0.05) (Fig. 1c-f).

### Toxic and side effects

Adverse reactions: the main adverse reactions due to chemotherapy were: (1) myelosuppression mainly manifested as leukopenia, anemia, and thrombocytopenia (30, 23, 21% in the test group vs 31, 22, 19% in the control group, respectively); (2) gastrointestinal reactions, including nausea, vomiting, and diarrhea (10, 15, 11% in the test group vs 13, 17, 11% in the control group, respectively); (3) abnormal renal and liver function (test group 33% vs control group 34%). It could be concluded that there was no significant difference in adverse reactions between the two groups (*P* > 0.05).

Complications: all patients were able to tolerate to the treatment, and only few patients showed oxygen desaturation, which could be relieved after the operation was stopped and oxygen inhalation was performed for 2–3 min. In the treatment of all the patients in the test group, no serious complications such as perforation of air tube wall, pneumothorax and massive hemorrhage were found, except for occasionally minor hemorrhage.

## Discussion

Lung cancer is a major global health threat, causing tens of thousands of deaths every year. The incidence of lung cancer is still high in many developing countries, particularly in East Asia [10]. Bronchoscope has become one of the most important tools for the diagnosis and treatment of pulmonary diseases, with applications ranging from airway evaluation to interventional treatment of airway lesions. With the introduction of new technologies, progress has been made in the use of two types of interventional bronchoscope, rigid and fiberoptic bronchoscope, to detect potentially life-threatening complications of advanced lung cancer, including airway obstruction and bleeding control [11]. Bronchoscopic interventional cryotherapy destroys endobronchial tumors and leads to tissue death through freezing tissue cytotoxic effects at extremely low temperatures (− 20 °C to − 40 °C) [12]. In addition, bronchoscopic interventional cryotherapy has been previously proved to only cause minor complications. It is also relatively convenient and economical compared to other treatments. Cryotherapy is safe, with no risk of bronchial wall perforation, radiation, electrical accidents or fire, and does not require much special training. The patients have a good tolerance to the operation and their symptoms can be obviously improved. With the improvement of medical level, the application of new cryosurgery probe makes the operation more convenient and safer [13]. This study aimed to explore the therapeutic effects of systemic chemotherapy combined with bronchoscopic interventional cryotherapy based on the advantages of cryotherapy. After this study, we mainly found that cryotherapy could improve the short-term effects and prolong the survival time of patients to a certain extent, meanwhile, it would not cause fatal complications and adverse reactions to patients.

Cryodamage is the main mechanism of cryotherapy for malignant tumors, and the success of cryotherapy is influenced by various factors. Studies have reported that cell survival depends on cooling rate [14], thawing rate [15], minimum temperature [16], repeated freezing and thawing cycle [17]. A cryosurgery probe is applied to the tissues to induce instant adherence of the probe to the tissues, followed by the appearance of ice crystals inside and outside the cells. These crystals destroy organelles in cells, especially mitochondria. The formation of pure extracellular ice crystals causes extra movement of ions and water, leading the cells to dehydrate. The greatest effect is achieved by rapidly freezing the tissues and then slowly thawing them [18]. In this study, all patients treated with systemic chemotherapy combined with bronchoscopic interventional cryotherapy obtained a good effect after treatment. Specifically, tumor ORR and DCR reached 76.9 and 91.3%, respectively, which were greatly improved compared with patients treated with systemic chemotherapy alone. Besides, the survival time of patients in the test group was also significantly longer, which was the most significant advantage of systemic chemotherapy combined with bronchoscopic interventional cryotherapy. In addition, after treatment, the levels of MMP and serum tumor markers in all patients were lower than those before treatment, and the decrease was more significant in patients in the test group. CD4^+^ and CD4^+^/CD8^+^ levels were also significantly higher in patients in the test group. Besides, we subdivided the types of lung cancer. The results exhibited that the effect of cryotherapy combined with chemotherapy in NSCLC patients was superior to that in SCLC patients, but there was no remarkable difference in the effect, MMP, serum tumor markers, T cell subsets level and survival time between LADC patients and LSCC patients. Zhikai et al. [19] found that the PFS of NSCLC patients (11 ± 5 months) was significantly better than that of SCLC patients (4 ± 2 months, *P* <  0.0001) in the treatment of 47 patients with central type lung cancer by using cryotherapy. The result is similar to our study. The reason for this phenomenon may be that the survival rate and metastatic ability of SCLC are generally higher than those of NSCLC [20, 21]. It is suggested that cryotherapy combined with systemic chemotherapy is more suitable for the treatment of NSCLC patients.

## Conclusion

In summary, interventional cryotherapy combined with chemotherapy has a good efficacy in the treatment of lung cancer. For patients with advanced lung cancer who are unable to undergo surgery, interventional cryotherapy provides an effective and safe treatment schedule, which can significantly improve the life quality and prolong the survival time of patients. Moreover, cryotherapy can also be used in other lung cancer treatments, such as the immunotherapy combining cryotherapy with PD-L1 inhibitor. In addition, bronchoscopic cryotherapy can also be applied to the initial treatment for patients with less severe disease. These schemes can be validated in future clinical trials. Overall, this study suggests that the clinical treatment of lung cancer can be combined with cryotherapy.

## Supplementary information

**Additional file 1: Figure S1.** Expression levels of MMP2 and MMP9 in peripheral blood of patients with different treatments. The expression levels of MMP2 and MMP9 in peripheral blood of patients in the test group and the control group (A-B), or of SCLC patients and NSCLC patients (C-D), or of LADC patients and LSCC patients (E-F) before and after treatment were detected by qRT-PCR. * means *P* <  0.05 and ns represents no significant difference.

**Additional file 2: Figure S2.** The content of CD4^+^, CD8^+^ and the ratio of CD4^+^/CD8^+^ in peripheral blood of patients in different treatment groups. Flow cytometry is used to detect the content of CD4^+^, CD8^+^ and the ratio of CD4^+^/CD8^+^ in peripheral blood of patients in the test group and the control group (A-C), or of SCLC patients and NSCLC patients (D-F), or of LADC and LSCC patients (G-I). * means *P* <  0.05 and ns indicates no significant difference.

## Data Availability

The datasets used and analysed during the current study are available from the corresponding author on reasonable request.

## Abbreviations

CA199: Carbohydrate antigen 199
CEA: Carcino-embryonic antigen
CR: Complete Remission
CYFRA21-1: Cytokeratin fragment antigen
DCR: Disease control rate
ELISA: Enzyme-linked immunosorbent assay
FCM: Flow cytometry
KPS: Karnofsky performance status
LADC: Lung adenocarcinoma
LSCC: Lung squamous cell carcinoma
MMP: Matrix metalloproteinase
NSCLC: Non-small cell lung cancer
NSE: Neuron-specific enolase
ORR: Objective response rate
OS: Overall survival
PD: Progressive Disease
PFS: progression-free survival
PR: Partial Remission
qRT-PCR: Real-time quantitative PCR
SCLC: Small-cell lung cancer
SD: Stable Disease
SFDA: State Food and Drug Administration
