# Supplementary figures and images for: Clinical study of systemic chemotherapy combined with bronchoscopic interventional cryotherapy in the treatment of lung cancer
Source: BMC Cancer. 2020 Nov 11;20:1089. doi: 10.1186/s12885-020-07444-6 (PMC7656695; doi:10.1186/s12885-020-07444-6)

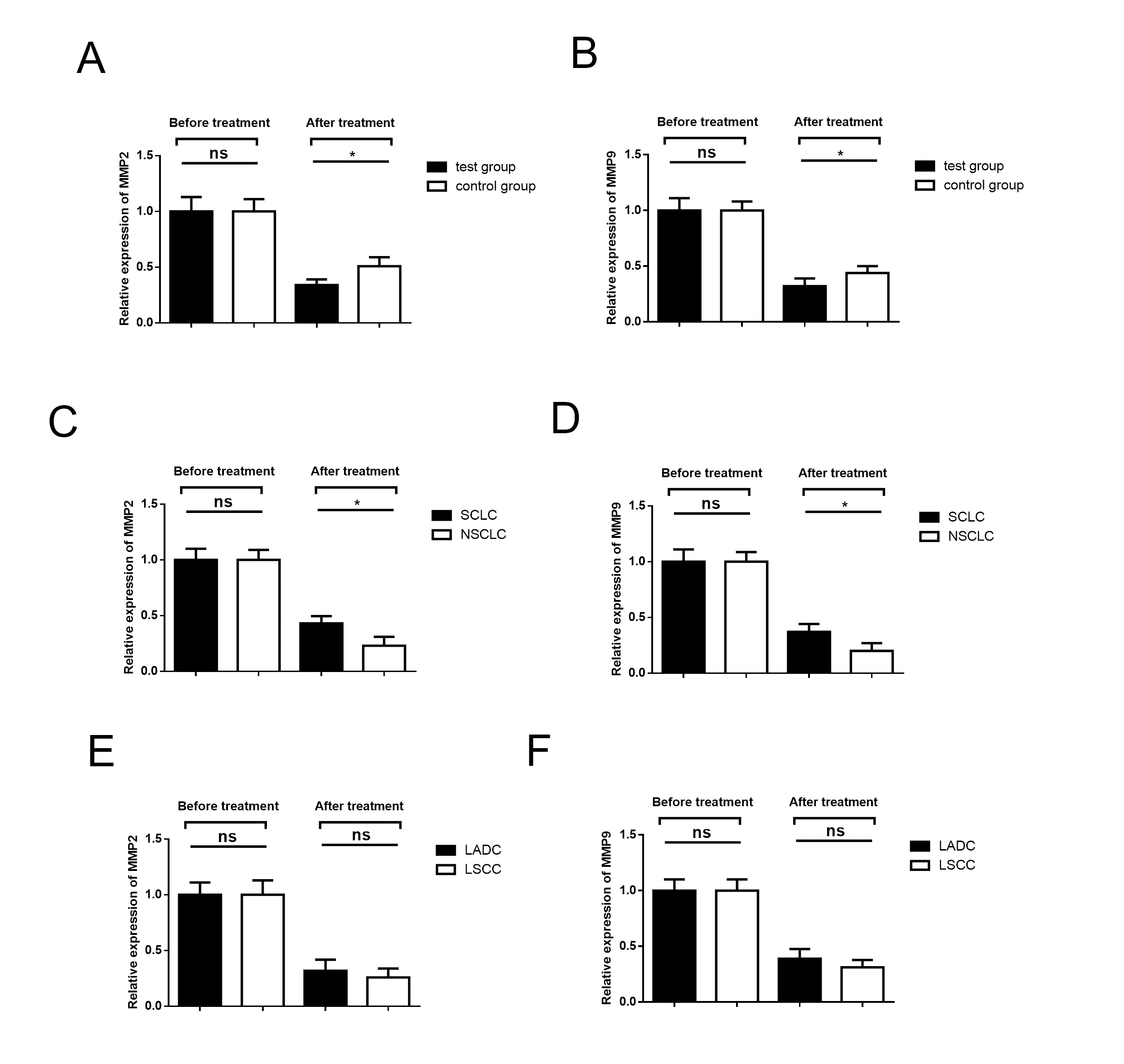

Supplement: Supplementary file 1 — Additional file 1: Figure S1. Expression levels of MMP2 and MMP9 in peripheral blood of patients with different treatments. The expression levels of MMP2 and MMP9 in peripheral blood of patients in the test group and the control group (A-B), or of SCLC patients and NSCLC patients (C-D), or of LADC patients and LSCC patients (E-F) before and after treatment were detected by qRT-PCR. * means P < 0.05 and ns represents no significant difference. [file 12885_2020_7444_MOESM1_ESM.tif]

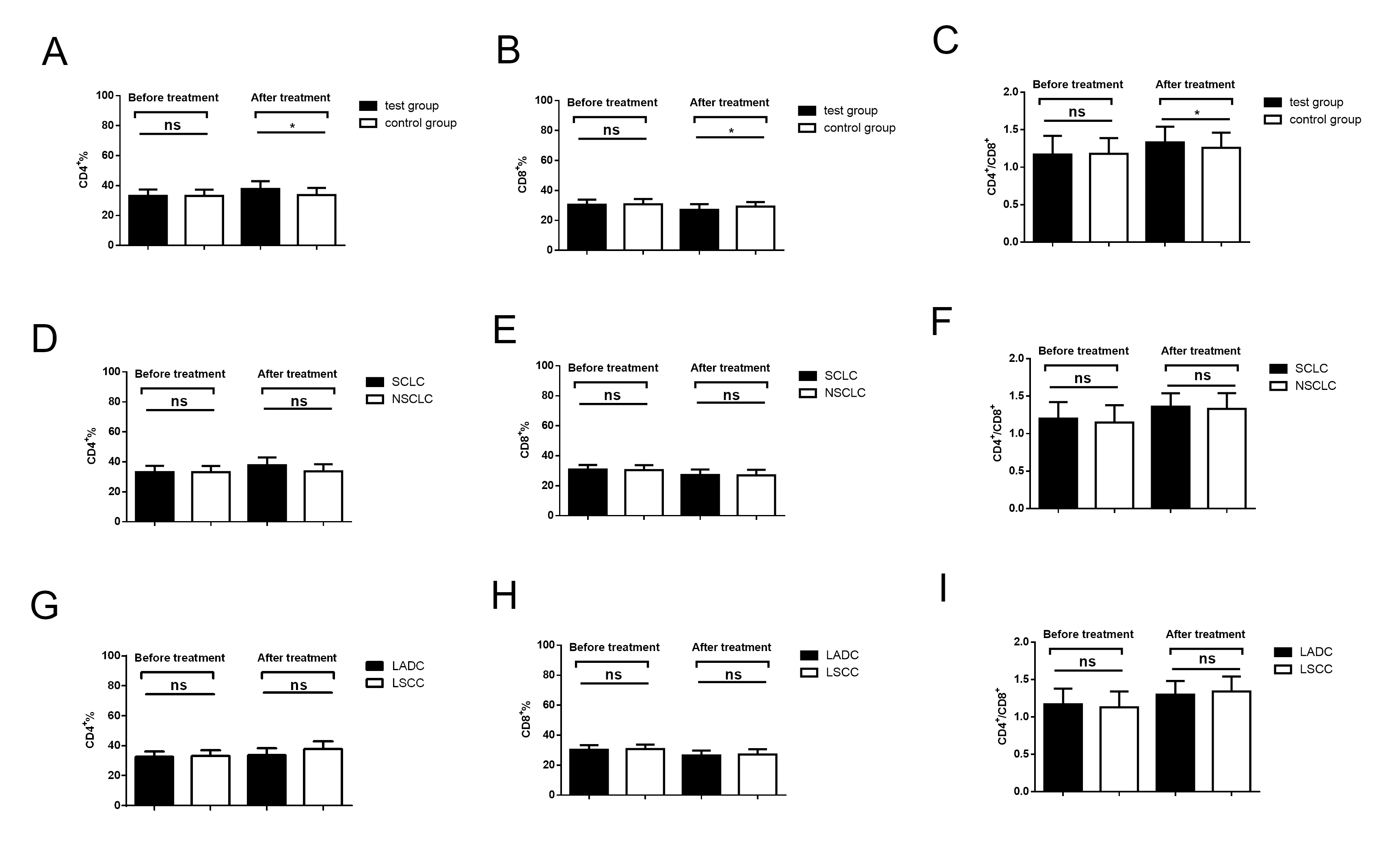

Supplement: Supplementary file 2 — Additional file 2: Figure S2. The content of CD4+, CD8+ and the ratio of CD4+/CD8+ in peripheral blood of patients in different treatment groups. Flow cytometry is used to detect the content of CD4+, CD8+ and the ratio of CD4+/CD8+ in peripheral blood of patients in the test group and the control group (A-C), or of SCLC patients and NSCLC patients (D-F), or of LADC and LSCC patients (G-I). * means P < 0.05 and ns indicates no significant difference. [file 12885_2020_7444_MOESM2_ESM.tif]
